# Chitinase 3‐like 1 is neurotoxic in multiple sclerosis patient‐derived cortical neurons

**DOI:** 10.1002/ctm2.70125

**Published:** 2024-12-10

**Authors:** Rucsanda Pinteac, Jordi Soriano, Clara Matute‐Blanch, José M Lizcano, Anna Duarri, Sunny Malhotra, Herena Eixarch, Gloria López Comellas, Xavier Montalban, Manuel Comabella

**Affiliations:** ^1^ Servei de Neurologia Centre d´Esclerosi Múltiple de Catalunya (Cemcat) Institut de Recerca Vall d´Hebron (VHIR) Hospital Universitari Vall d´Hebron Universitat Autònoma de Barcelona (UAB) Barcelona Spain; ^2^ Center for Networked Biomedical Research on Neurodegenerative Diseases (CIBERNED) ‐ ISCIII Madrid Spain; ^3^ Departament de Física de la Matèria Condensada, Facultat de Física Universitat de Barcelona Barcelona Spain; ^4^ Universitat de Barcelona Institute of Complex Systems (UBICS) Universitat de Barcelona Barcelona Spain; ^5^ Protein Kinases in Cancer Research Vall d'Hebron Institut de Recerca (VHIR) Barcelona Spain; ^6^ Departament de Bioquímica i Biologia Molecular and Institut de Neurociències Facultat de Medicina Universitat Autònoma de Barcelona (UAB) Barcelona Spain; ^7^ Ophthalmology Research Group Vall d'Hebron Institut de Recerca Barcelona Spain

1

Dear Editor,

We are pleased to present our latest findings regarding the neurotoxic role of Chitinase 3‐like 1 (CHI3L1) in multiple sclerosis (MS). CHI3L1, a 40 kD glycoprotein, is primarily produced by activated astrocytes and microglia in the central nervous system (CNS), and it has garnered considerable attention due to its implications in inflammation and tissue remodelling.^1^ It is notably increased in several conditions, including MS, and accumulating evidence supports CHI3L1 as a biomarker in early MS, with elevated cerebrospinal fluid (CSF) levels associated with increased disability risk.^2^, ^3^ This association led us to investigate whether CHI3L1 simply reflects glial activation or if it exerts direct neurotoxicity. Our prior work in murine neurons demonstrated CHI3L1's neurotoxic effects,^4^ prompting us to explore its impact on MS patient‐derived human induced pluripotent stem cells (hiPSC). Here, we aim to characterize these effects at both molecular and functional levels, further exploring CHI3L1's potential as a biomarker and therapeutic target for MS.

The first step in our investigation involved refining a human neuronal model using two MS‐derived hiPSC lines (Table S1), MS‐10 and MS‐6, matured for 28 and 40 days (Figure S1A). To ensure the model's suitability, we meticulously characterized the neuronal cultures through immunofluorescence and calcium imaging, evaluating neuronal and astrocytic proportions (Figure S1B–E,J,L), cortical fate (Figure S1F–I,K,M), dendrite growth (Figure S2A), synaptic (Figure S2B) and neurotransmitter markers (Figure S3) and the onset of sporadic and synchronous neuronal activity (Figure S4). The neuronal cultures exhibited varying percentages of astrocytes, which increased over time for both cell lines (Figure S1J,L). Cortical fate was delineated by robust Tbr1 immunoreactivity alongside limited CTIP2 expression (Figure S1K,M). Notably, dendritic growth persisted until day 28 (Figure S2A), coinciding with the expression of synaptic markers like synapsin and PSD‐95 (Figure S2B). By day 28, most neurons from each line co‐expressed glutamatergic and GABAergic markers, but by day 40, the loss of vGAT immunoreactivity suggested a predominant population of glutamatergic cells (Figure S3). Additionally, fluorescence calcium imaging revealed a progressive increase in spontaneous and synchronous neuronal activity over time, culminating in a sporadic and synchronous pattern by day 40 (Figure S4).

Our investigation progressed to examine the impact of CHI3L1 on neuronal morphology and synaptic plasticity by treating MS‐10 and MS‐6 neuronal cultures at day 28 with CHI3L1 (300 ng/mL) or vehicle (PBS) for 24 and 72 h (Figure 1A). The analysis revealed a 17.5% reduction in dendritic arborization by 24 h and a 19% reduction by 72 h, along with a 16.5% decrease in dendrite length at 72 h (Figure 1B–D). Additionally, synaptic plasticity assessment unveiled CHI3L1‐induced decreases in synapsin area (23.3%) and active synapses (47.9%) at 72 h, accompanied by a trend towards decreased PSD‐95 levels (Figure 1E–H), indicating compromised structural integrity and synaptic function. The CHI3L1‐induced reductions in dendritic arborization and synaptic connectivity are consistent with hallmark features of neurodegenerative diseases, including MS.

**FIGURE 1:**
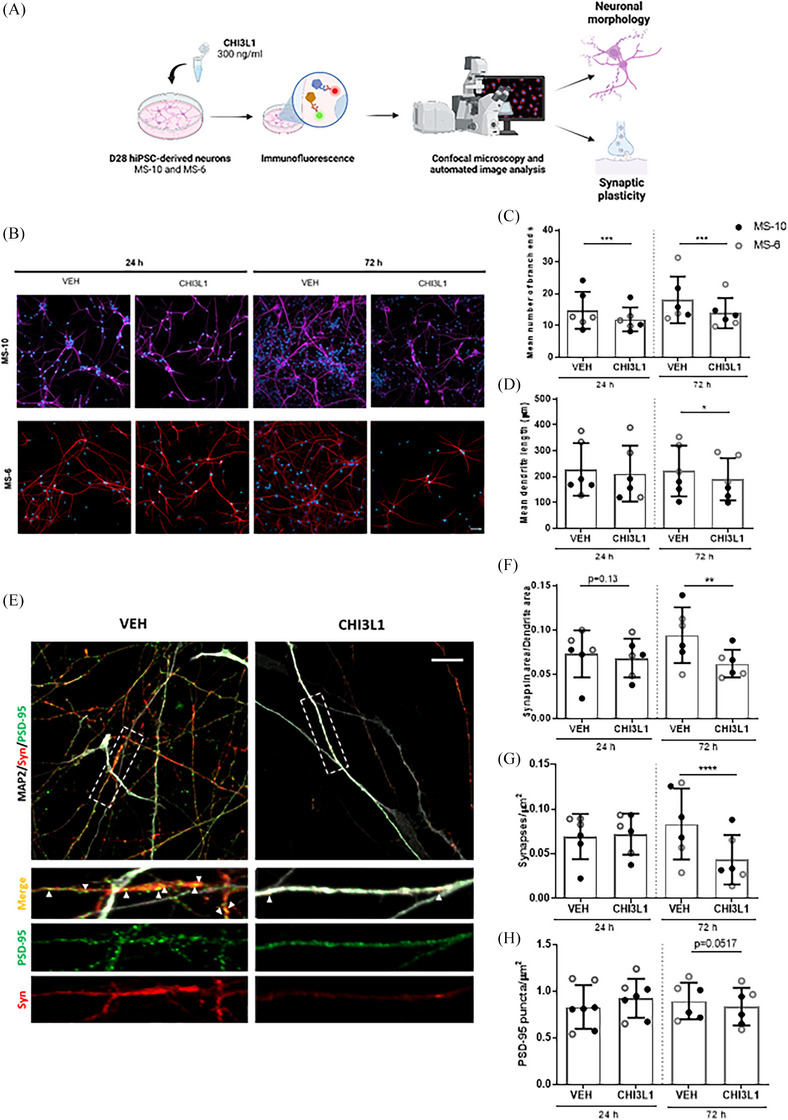
Chitinase 3‐like 1 (CHI3L1) induces morphological and synaptic alterations in human induced pluripotent stem cells (hiPSC)‐derived neuronal cultures. (A) Workflow of the morphological and synaptic study. For the morphological and synaptic study, we treated neuronal cultures derived from MS‐10 and MS‐6 cell lines at day 28 of maturation with CHI3L1 (300 ng/mL) or vehicle (PBS) for 24 and 72 h. For morphological analysis, we performed immunofluorescence to label MAP2, a dendritic marker, allowing visualization of dendritic structures, and DAPI, a nuclear stain. Total dendrite length and the number of branch ends were measured to evaluate dendritic complexity. Synaptic analysis involved co‐labeling MAP2 with synapsin, a presynaptic marker, and PSD‐95, a postsynaptic marker, to evaluate synaptic compartments and identify active synapses, defined by the co‐localization of synapsin and PSD‐95 within dendritic structures. (B) Representative immunofluorescence images from the morphological study, where MAP2 is visualized in magenta or red and DAPI in blue. Scale bar = 50 µM. (C, D) Graphs depict the number of branch ends and the mean dendrite length, respectively. Mean values for dendrite length and the number of branch ends were obtained for each image. Statistical analysis was performed on mean image values (155–191 images per condition) from both cell lines. (E) Representative immunofluorescence images from the synaptic study at 72 h after CHI3L1 or vehicle (VEH) treatment, where MAP2 is visualized in white, the presynaptic marker synapsin in red, and the postsynaptic marker PSD‐95 in green. Scale bar = 10 µM. Co‐localization of synapsin and PSD‐95 indicated active synapses. Automated quantitative analysis was performed on images from both cell lines (164‐202 images per condition). (F–H) Statistical analysis was carried out on mean image values from both cell lines. Graphs depict synapsin area/dendrite area (F), and synapse density (G) and PSD‐95 puncta density (H). Graph bars represent mean ± SD. Black and grey dots indicate the mean of independent experiments (*n = *6) performed with MS‐10 (*n = *3) and MS‐6 (*n = *3) lines, respectively. *p < .05; **p < .01; ***p < .001; (one‐way analysis of variance [ANOVA] with Sidak's post hoc).

We then investigated the impact of CHI3L1 on neuronal and population activity. MS‐10 and MS‐6 neuronal cultures at day 40 were treated with CHI3L1 (300 ng/mL) or vehicle (PBS) for 4, 24 and 72 h (Figure 2A). Using fluorescent calcium imaging, we monitored neuronal activity and applied advanced computational techniques^5^ for analysis. Our results revealed a notable increase in fluorescence amplitude within CHI3L1‐treated cultures, which was only significant at the 4‐h mark (Figure 2B), indicative of heightened excitability. While no significant differences were observed in the percentage of active neurons (Figure 2C), mean neuronal activity (Figure 2D) or the inter‐burst interval (IBI; Figure 2E), our network behaviour analysis unveiled dynamic shifts over time. Initially, there was an increase in the strength of collective events (SCE), followed by a gradual decline at 24 and 72 h (Figure 2F). Although our assessment of effective connectivity did not reveal significant differences, the observed trends suggest altered network integration dynamics over time (Figure 2G–I), mirroring the shifts noted in collective behaviour.

**FIGURE 2:**
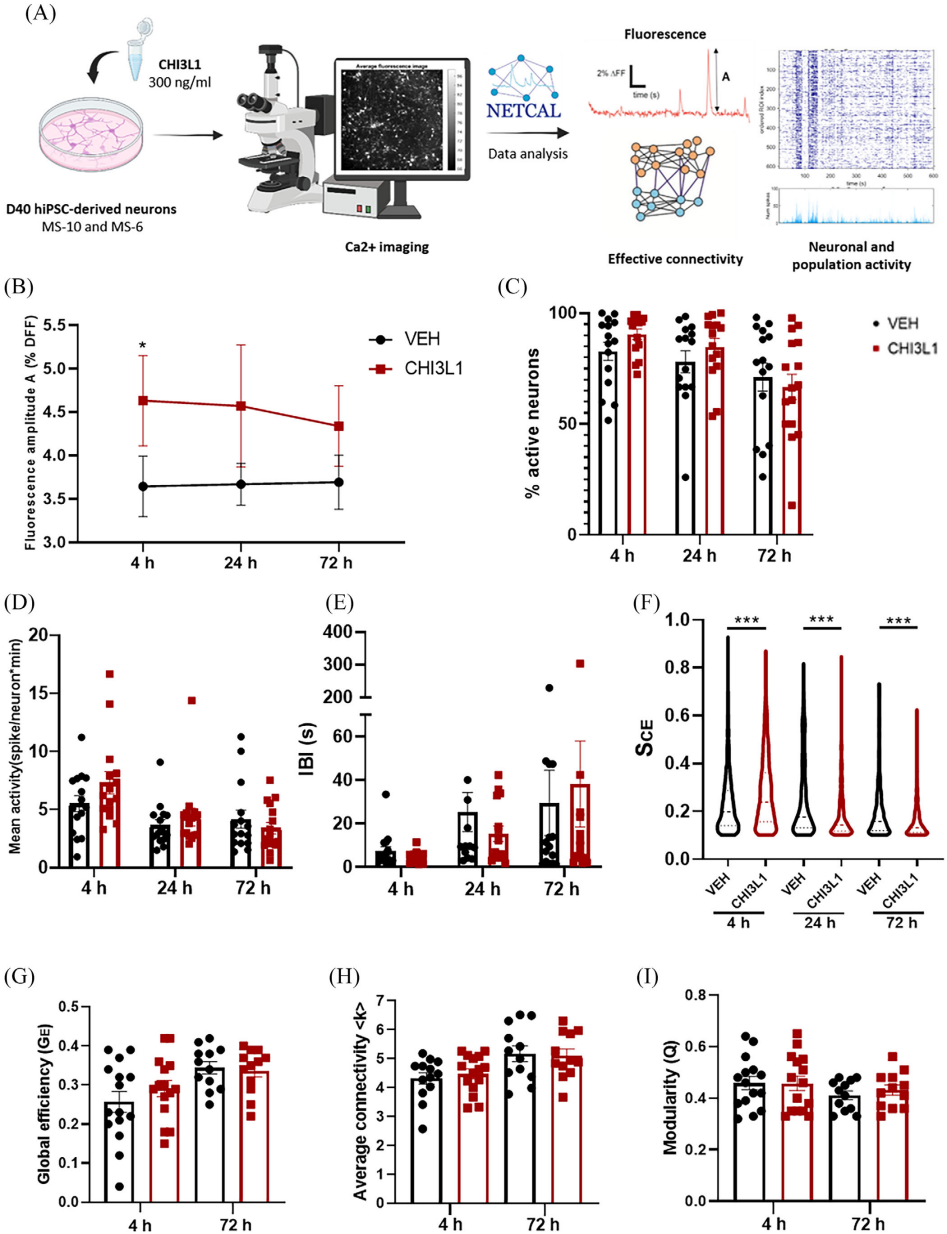
Chitinase 3‐like 1 (CHI3L1) increases neuronal excitability and induces dynamic population activity changes. (A) Workflow of the neuronal and population activity and effective connectivity studies. Neuronal cultures derived from MS‐10 and MS‐6 cell lines at day 40 of maturation were treated with CHI3L1 (300 ng/mL) or vehicle (VEH, PBS) for 4, 24 and 72 h. Neuronal activity was assessed using fluorescent calcium imaging to monitor calcium transients, indicative of neuronal firing, via a calcium‐binding dye. The acquired data were analyzed using the NETCAL software. Fluorescence recordings were pre‐processed to remove background noise, and neurons were selected as regions of interest (ROIs)—automatically for MS‐10 and manually for MS‐6. Traces were extracted as ROI‐average signals over time, with sharp fluorescence increases representing neuronal activations. The detection algorithm produced raster plots to visualize the spatiotemporal activity of the neuronal network. The ROIs centres, fluorescence traces, and neuronal spikes were analyzed to extract key parameters of network dynamics, including average neuronal activity, the strength of collective events (SCE), inter‐burst intervals (IBI), the percentage of active neurons, mean culture activity, global efficiency, and average connectivity, using advanced computational methods. (B) CHI3L1 increased the fluorescence amplitude of the neuronal cultures. Data are shown as mean ± SEM. (C‐E, G‐I) Data are shown as mean ± SEM for various parameters: percentage of active neurons (C), mean culture activity (D), IBI (E), global efficiency (G), modularity (H) and average connectivity (I). (F) CHI3L1 induced dynamic changes in the population activity, specifically in the SCE. Violin plots depict the distribution of the SCE, indicating the proportion of the network involved in collective events, with median and quartiles represented by continuous and dashed lines, respectively. (B–I) Six independent experiments were conducted, three using each cell line (MS‐10 and MS‐6). Dots in (C–E, G–I) represent the mean value of individual replicates from six independent experiments. *p < .05, ***p < .001; (one‐way ANOVA with Sidak's post hoc).

Following the characterization of CHI3L1‐induced neurotoxic effects, we investigated the underlying molecular mechanisms. Neuronal cultures derived from MS‐10 cells at day 28 were treated with CHI3L1 (300 ng/mL) or vehicle (PBS) for 12 and 24 h, followed by gene expression analysis using microarrays. This analysis identified numerous differentially expressed genes (DEGs) linked to neurodegenerative disorders and synaptic activity (Tables S2–5). Seven DEGs (RIOK2, DENND2C, CFAP61, RASA2, LRRC66, UHMK1 and GNMT) were validated through quantitative real‐time polymerase chain reaction (Figure 3A). Notably, some of the validated DEGs, such as RIOK2,^6^ have been reported to be involved in neurodegenerative disorders, while others like UHMK1^7^ and GNMT^8^ play roles in neurite growth and neurogenesis. Functional analysis revealed enrichment in categories related to receptor‐ligand activity, signalling receptor activator activity and proinflammatory processes at 12 h (Figure 3B), transitioning to synaptic activity‐related processes at 24 h (Figure 3C), as confirmed by Gene Set Enrichment Analysis (GSEA) (Figure 3D). These findings underscore CHI3L1's impact on genes and pathways critical to neuronal function and neurodegeneration, particularly at the 24‐h mark.

**FIGURE 3:**
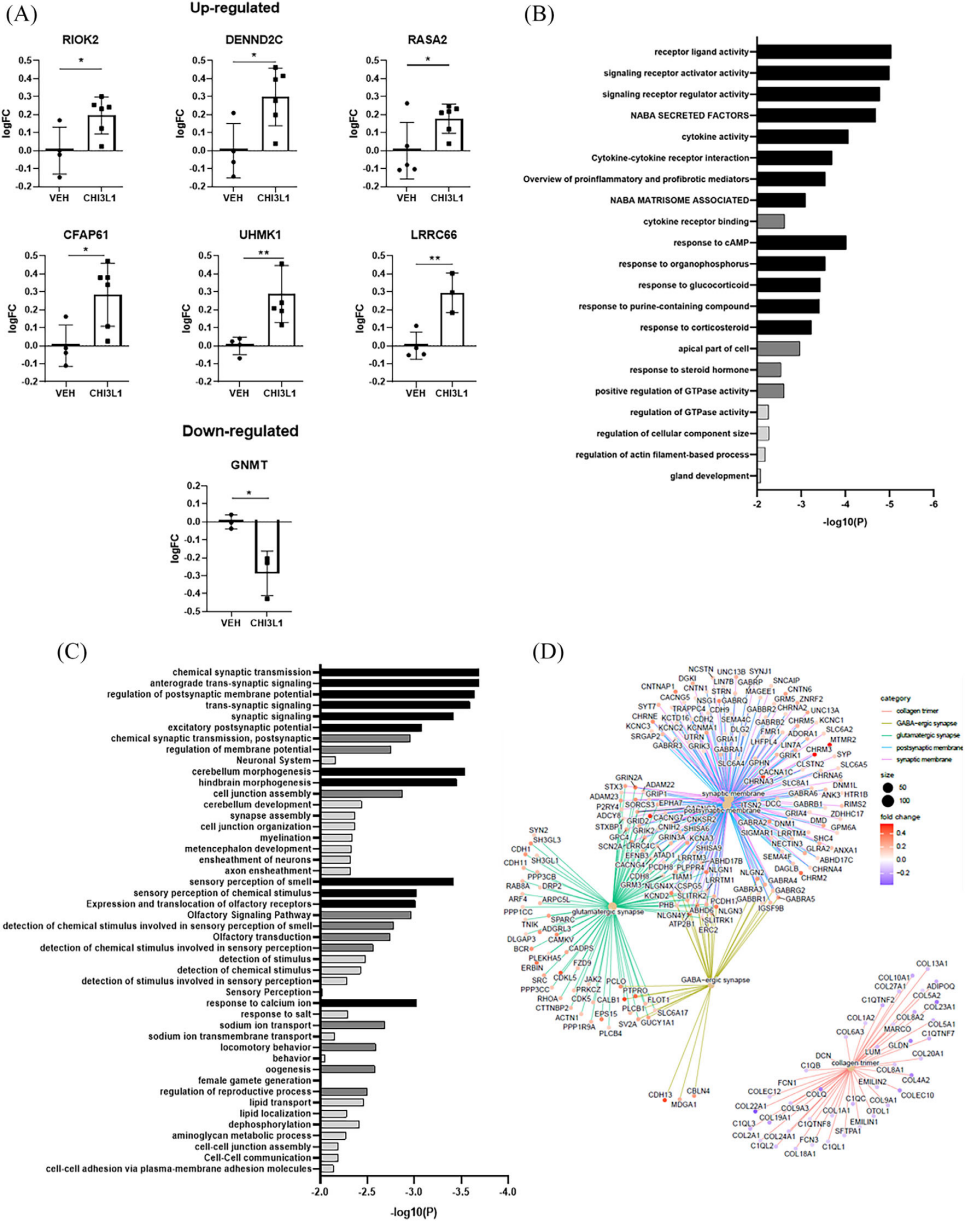
The Pleiotropic influence of Chitinase 3‐like 1 (CHI3L1) extends to diverse molecular events, influencing pathways and genes associated with inflammatory and neurodegenerative processes. (A) Validation of differentially expressed genes (DEGs) at CHI3L1 24 h treatment by quantitative real‐time polymerase chain reaction (RT‐qPCR). mRNA expression levels were measured by RT‐qPCR and quantified using the 2‐ΔΔCt method. Individual values represent logFC of mRNA expression comparing CHI3L1 versus vehicle (VEH). n = 3‐6 independent experiments per group. Graphs show RT‐qPCR analysis of RIOK2, DENND2C, RASA2, CFAP61, UHMK1, LRRC66 and GNMT mRNA expression. Data are presented as mean ± SD. (B) Functional enrichment analysis of upregulated DEGs at CHI3L1 12 h treatment. (C) Functional enrichment analysis of upregulated DEGs at 24 h DEGs. (D) GSEA analysis. Bars in Figures 3B and 3D are colour‐coded to indicate three levels of statistical significance: the darkest bars represent the most significant changes, the light grey bars represent intermediate significance, and the lightest grey bars represent the least significant changes. *p < .05, **p < .01; (two‐tailed unpaired t‐test). CFAP61 ‐ Cilia and flagella associated protein 61, DEGs—differentially expressed genes, DENND2C—DENN domain‐containing 2C, GNMT—Glycine N‐Methyltransferase, GSEA—gene set enrichment analysis, LRRC66 ‐ Leucine‐rich repeat containing 66, RASA2 ‐ RAS P21 protein activator 2, RIOK2 ‐ RIO kinase 2 and UHMK1 ‐ U2AF homology motif kinase 1.

In parallel, we conducted a protein phosphorylation array analysis to explore the signalling pathways underlying CHI3L1's neurotoxicity. MS‐10‐derived neuronal cultures exposed to CHI3L1 (300 ng/mL) or vehicle (PBS) at day 28 were analyzed for key protein phosphorylation levels at early time points. We observed consistent increases in the phosphorylation levels of STAT1, particularly at Y701 (Figure 4A). Immunoblot analyses confirmed STAT1‐Y701 phosphorylation at 2 h post‐exposure (Figure 4B). TRRUST enrichment analysis identified IRF1 and STAT1 as potential transcription factors governing the gene expression response to CHI3L1 treatment at 24 h, implicating the interferon response pathway (Figure 4C). Comparison with the Transcription Factor Target Gene Database supported the involvement of IRF1 and STAT1 in mediating CHI3L1's effects on hiPSC‐derived neurons (Figure 4D).

**FIGURE 4:**
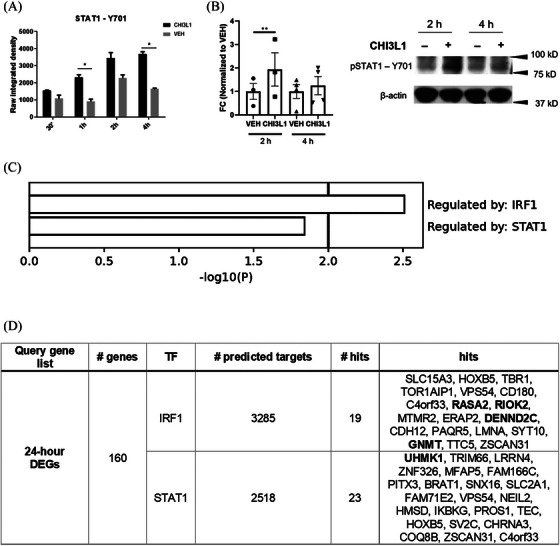
The interferon response pathway may mediate Chitinase 3‐like 1 (CHI3L1)‐induced neurotoxic effects. (A) Graphs showing integrated pixel density values for STAT1‐Y701 phosphorylation, assessed using the Human Phospho‐Kinase Array (R&D Systems). Data are derived from two technical replicates per condition at each time point (30 min, 1 h, 2 h and 4 h). Statistical analysis was performed using a two‐way ANOVA with Greenhouse‐Geisser correction, followed by Bonferroni post hoc tests to compare CHI3L1‐treated versus vehicle‐treated conditions at each time point. Data are presented as mean ± SD. *p < .05. (B) Western blot validation of STAT1‐Y701 phosphorylation in human induced pluripotent stem cells (hiPSC)‐derived neuronal cultures treated with CHI3L1. Left: graph showing the fold change in STAT1‐Y701 phosphorylation relative to vehicle‐treated cultures, normalized to vehicle controls. Data are presented as mean ± SD, with each dot representing an independent experiment (*n* = 3–4). Right: representative immunoblots for STAT1‐Y701 phosphorylation (80 kDa) and β‐actin (42 kDa) as an endogenous control. Statistical analysis was conducted using a two‐tailed paired t‐test. **p < .01. (C) Summary of TRRUST transcription factor enrichment analysis performed with Metascape. (D) Transcription Factor Target Gene Database comparative analysis of 24‐h differentially expressed genes (DEGs) against IRF1 and STAT1 targets. Bold entries indicate RT‐qPCR‐validated genes. BRAT1 ‐ BRCA1‐associated ATM activator 1, CD180 ‐ CD180 molecule, CDH12 ‐ Cadherin 12, C4orf33 ‐ Chromosome 4 open reading frame 33, CHRNA3 ‐ Cholinergic receptor nicotinic alpha 3 subunit, COQ8B—Coenzyme Q8B, DEGs—differentially expressed genes, DENND2C—DENN domain containing 2C, ERAP2 ‐ Endoplasmic reticulum aminopeptidase 2, FAM166C—Family with sequence similarity 166 member C, FAM71E2 ‐ Family with sequence similarity 71 member E2, GNMT—Glycine N‐methyltransferase, HMSD—Histocompatibility (minor) serpin domain containing, HOXB5 ‐ Homeobox B5, IKBKG—Inhibitor of nuclear factor kappa B kinase regulatory subunit gamma, LRRN4 ‐ Leucine‐rich repeat neuronal 4, LMNA—Lamin A/C, MFAP5 ‐ Microfibril‐associated protein 5, MTMR2 ‐ Myotubularin related protein 2, NEIL2 ‐ Nei endonuclease VIII‐like 2, PAQR5 ‐ Progestin and adipoQ receptor family member V, PITX3 ‐ Paired‐like homeodomain 3, PROS1 ‐ Protein S, RASA2 ‐ RAS p21 protein activator 2, RIOK2 ‐ RIO kinase 2, SLC15A3 ‐ Solute carrier family 15 member 3, SLC2A1 ‐ Solute carrier family 2 member 1, SNX16 ‐ Sorting nexin 16, SV2C—Synaptic vesicle glycoprotein 2C, SYT10 ‐ Synaptotagmin 10, TBR1 ‐ T‐box brain transcription factor 1, TEC—Tec protein tyrosine kinase, TF‐ transcription factor, TOR1AIP1 ‐ Torsin A interacting protein 1, TRIM66 ‐ Tripartite motif containing 66, TTC5 ‐ Tetratricopeptide repeat domain 5, UHMK1 ‐ U2AF homology motif kinase 1, VPS54 ‐ VPS54 subunit of GARP complex, ZNF326 ‐ Zinc finger protein 326, ZSCAN31 ‐ Zinc finger and SCAN domain containing 31 and TF—transcription factor.

The elucidation of neurodegenerative processes in MS is vital for targeted therapeutic strategies. In this study, we observed neurotoxic effects on dendritic morphology, synaptic function and neuronal excitability, indicative of its potential as an MS prognostic biomarker. Transcriptomic analyses unveiled a complex signature involving pathways and genes related to inflammation and synaptic function, alongside activation of STAT1 post‐CHI3L1 treatment. Understanding its intricate molecular mechanisms may unveil new therapeutic targets for inhibiting CHI3L1‐mediated neuronal signalling, offering promising avenues for targeted interventions in MS.

## Supporting information



Supporting information

Supporting information
