# Supplementary material for: Chitinase 3‐like 1 is neurotoxic in multiple sclerosis patient‐derived cortical neurons
Source: Clin Transl Med. 2024 Dec 10;14(12):e70125. doi: 10.1002/ctm2.70125 (PMC11631566; doi:10.1002/ctm2.70125)
Supplement: Supplementary file 1 — Supporting information [file CTM2-14-e70125-s001.pptx]

## Slide 1
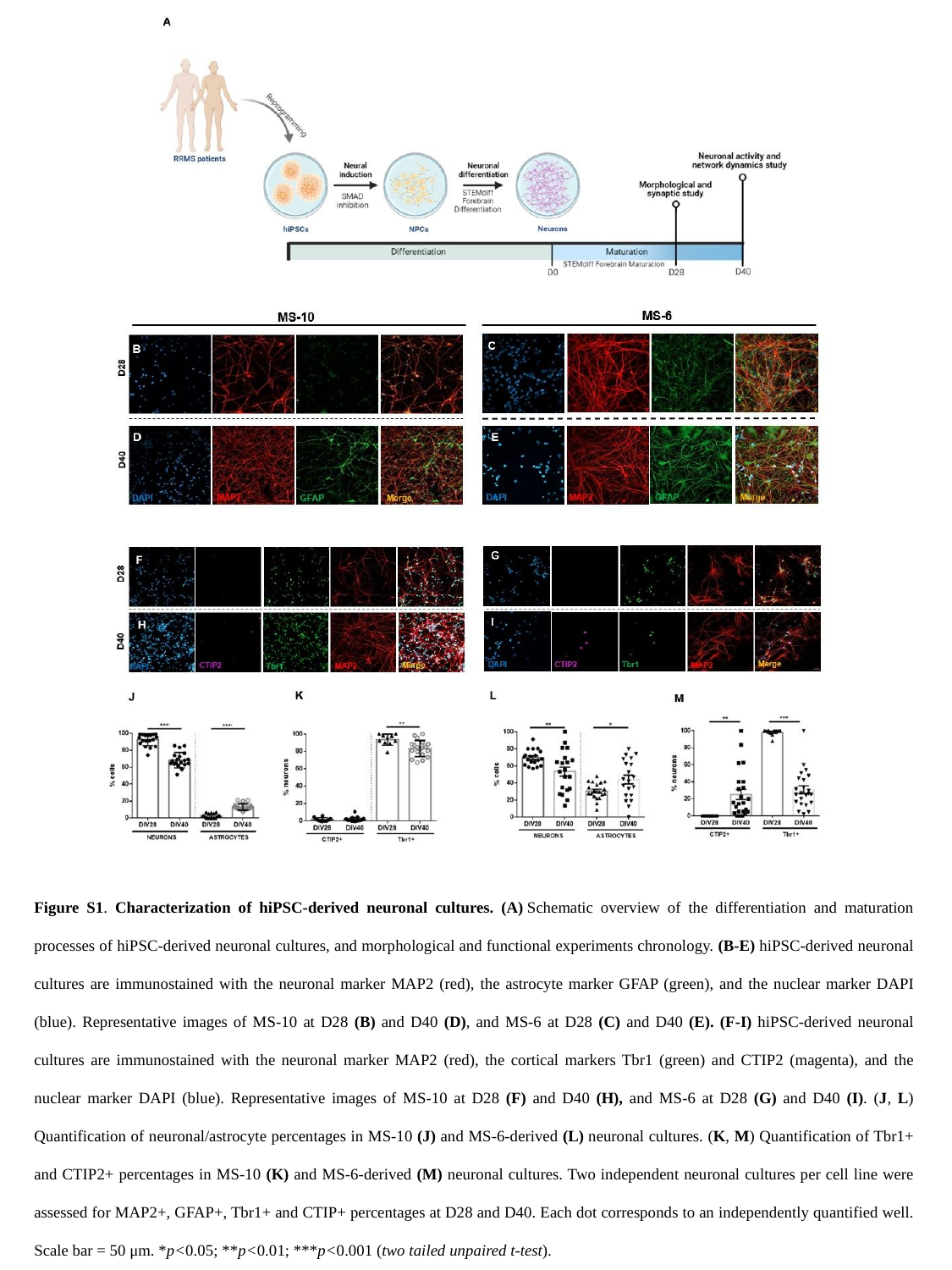

Figure S1. Characterization of hiPSC-derived neuronal cultures. (A) Schematic overview of the differentiation and maturation processes of hiPSC-derived neuronal cultures, and morphological and functional experiments chronology. (B-E) hiPSC-derived neuronal cultures are immunostained with the neuronal marker MAP2 (red), the astrocyte marker GFAP (green), and the nuclear marker DAPI (blue). Representative images of MS-10 at D28 (B) and D40 (D), and MS-6 at D28 (C) and D40 (E). (F-I) hiPSC-derived neuronal cultures are immunostained with the neuronal marker MAP2 (red), the cortical markers Tbr1 (green) and CTIP2 (magenta), and the nuclear marker DAPI (blue). Representative images of MS-10 at D28 (F) and D40 (H), and MS-6 at D28 (G) and D40 (I). (J, L) Quantification of neuronal/astrocyte percentages in MS-10 (J) and MS-6-derived (L) neuronal cultures. (K, M) Quantification of Tbr1+ and CTIP2+ percentages in MS-10 (K) and MS-6-derived (M) neuronal cultures. Two independent neuronal cultures per cell line were assessed for MAP2+, GFAP+, Tbr1+ and CTIP+ percentages at D28 and D40. Each dot corresponds to an independently quantified well. Scale bar = 50 μm. *p<0.05; **p<0.01; ***p<0.001 (two tailed unpaired t-test).

## Slide 2
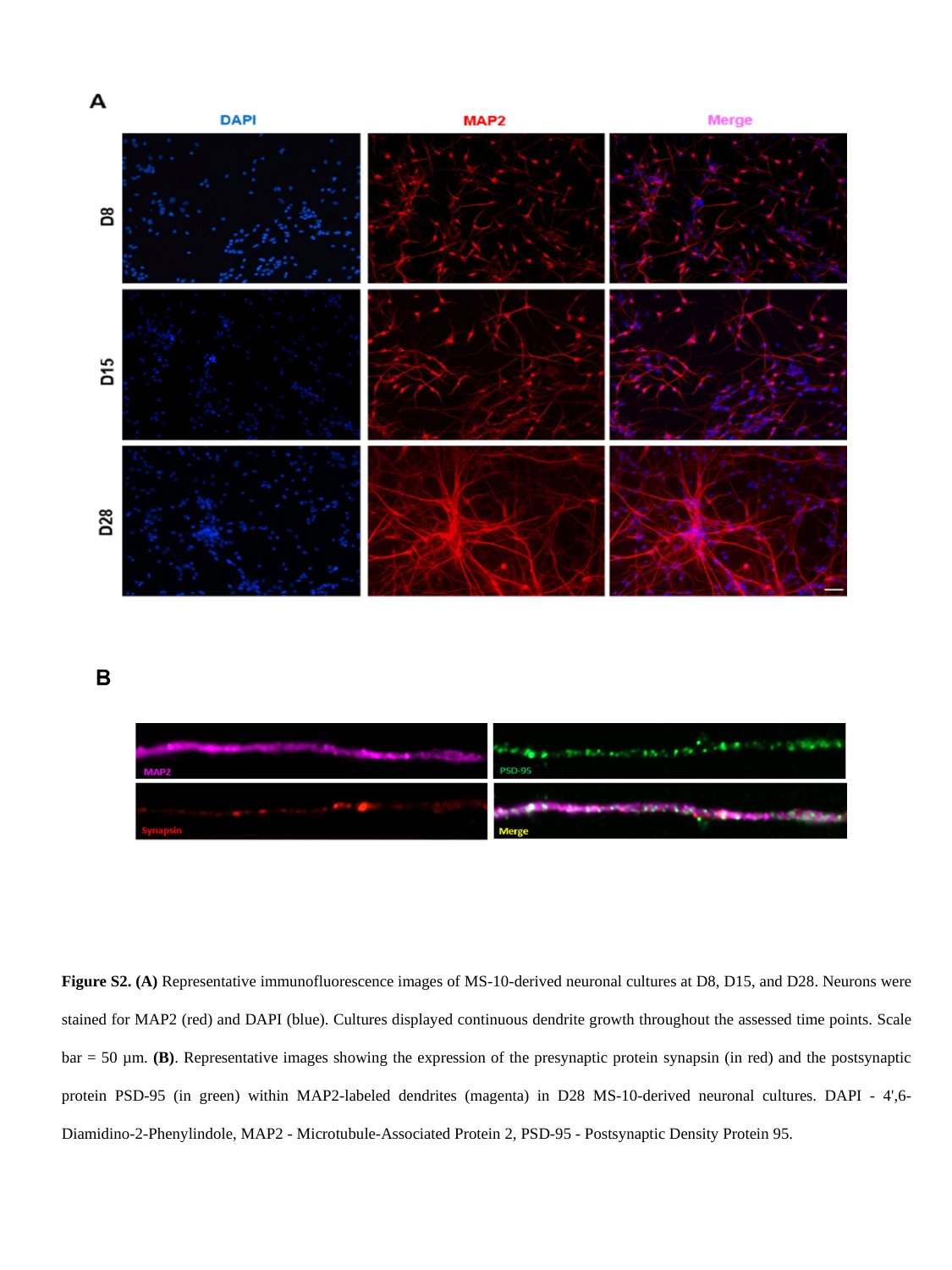

Figure S2. (A) Representative immunofluorescence images of MS-10-derived neuronal cultures at D8, D15, and D28. Neurons were stained for MAP2 (red) and DAPI (blue). Cultures displayed continuous dendrite growth throughout the assessed time points. Scale bar = 50 µm. (B). Representative images showing the expression of the presynaptic protein synapsin (in red) and the postsynaptic protein PSD-95 (in green) within MAP2-labeled dendrites (magenta) in D28 MS-10-derived neuronal cultures. DAPI - 4',6-Diamidino-2-Phenylindole, MAP2 - Microtubule-Associated Protein 2, PSD-95 - Postsynaptic Density Protein 95.

## Slide 3
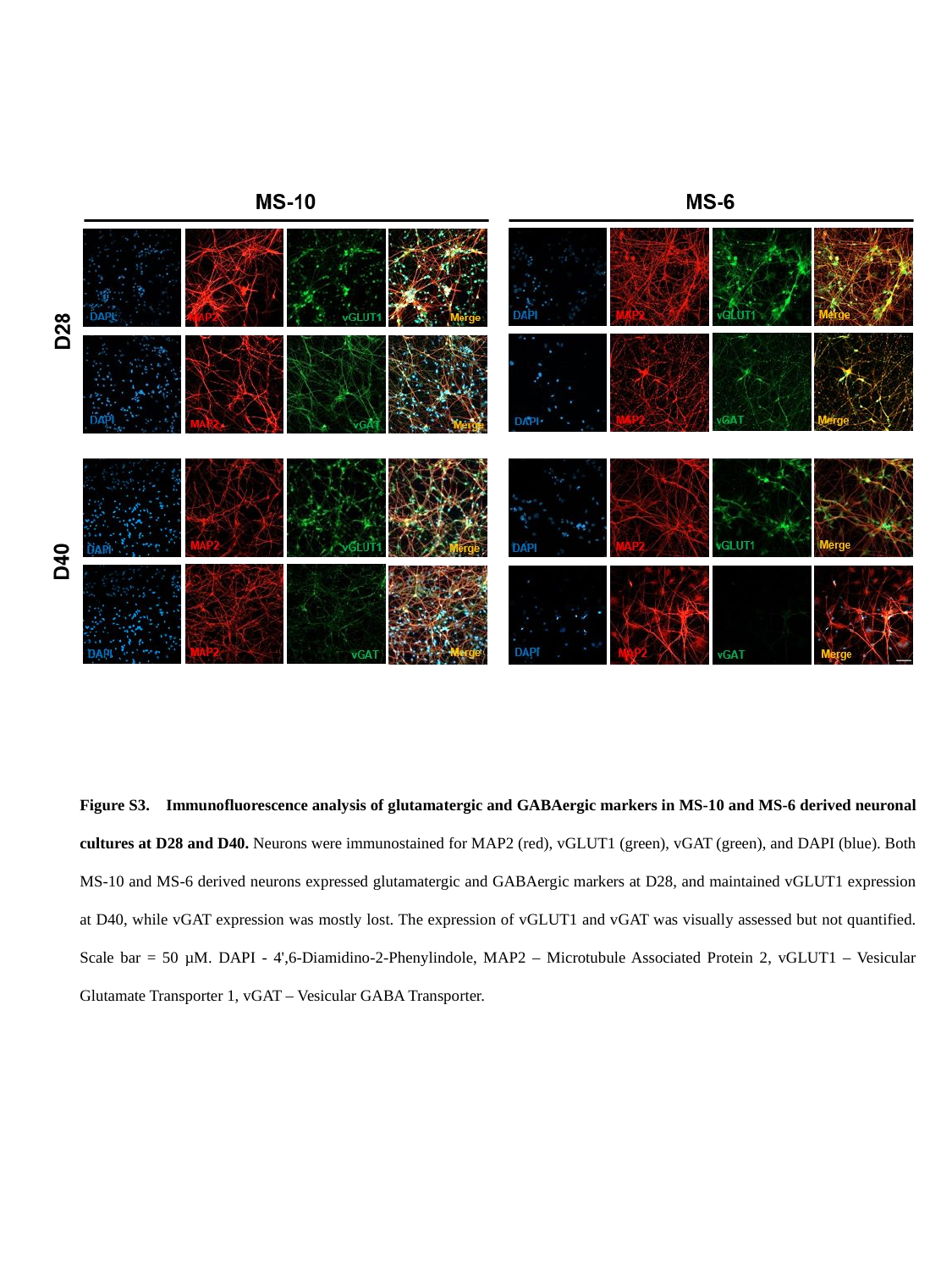

Figure S3.    Immunofluorescence analysis of glutamatergic and GABAergic markers in MS-10 and MS-6 derived neuronal cultures at D28 and D40. Neurons were immunostained for MAP2 (red), vGLUT1 (green), vGAT (green), and DAPI (blue). Both MS-10 and MS-6 derived neurons expressed glutamatergic and GABAergic markers at D28, and maintained vGLUT1 expression at D40, while vGAT expression was mostly lost. The expression of vGLUT1 and vGAT was visually assessed but not quantified. Scale bar = 50 µM. DAPI - 4',6-Diamidino-2-Phenylindole, MAP2 – Microtubule Associated Protein 2, vGLUT1 – Vesicular Glutamate Transporter 1, vGAT – Vesicular GABA Transporter.

## Slide 4
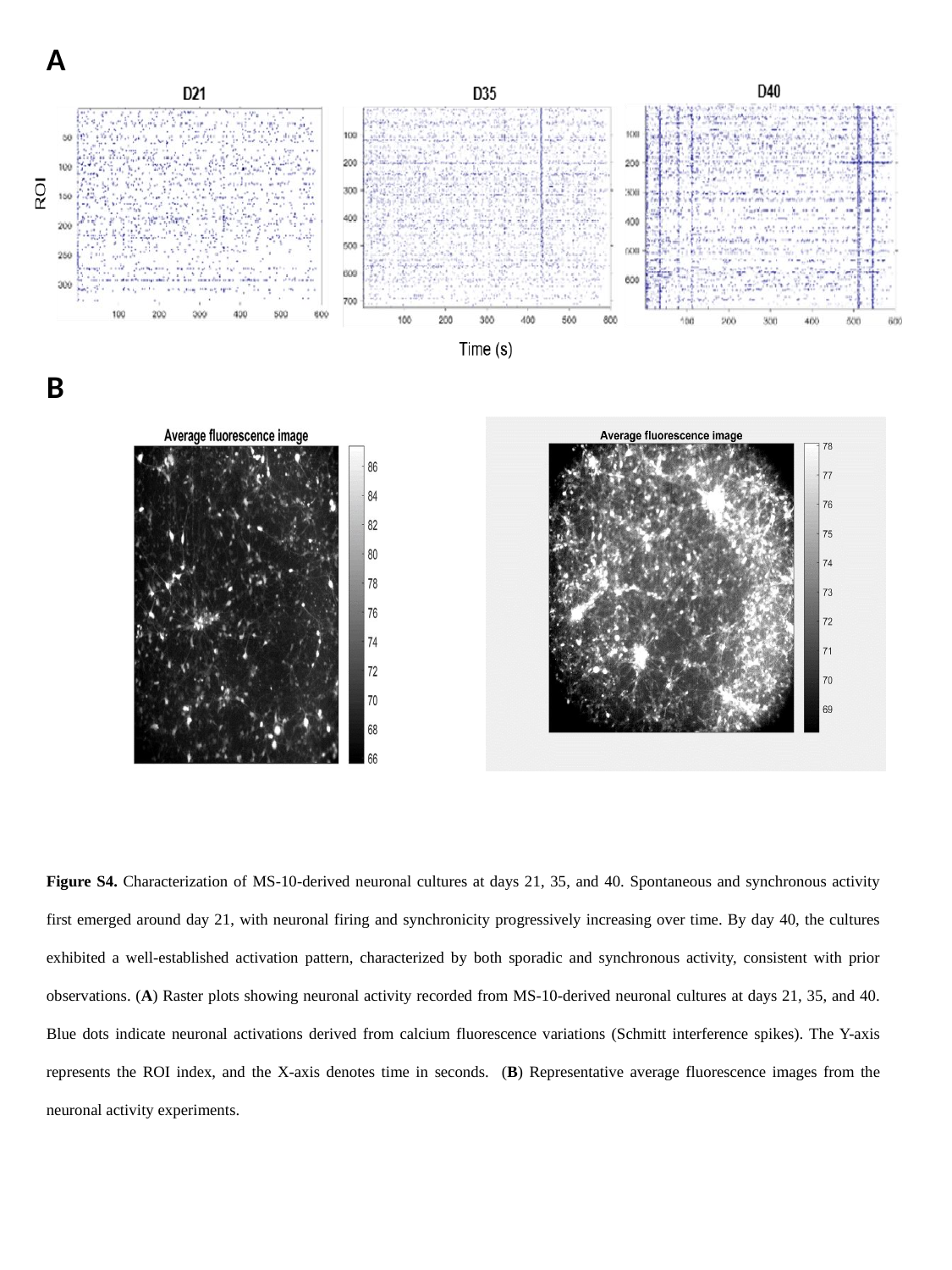

A
B
Figure S4. Characterization of MS-10-derived neuronal cultures at days 21, 35, and 40. Spontaneous and synchronous activity first emerged around day 21, with neuronal firing and synchronicity progressively increasing over time. By day 40, the cultures exhibited a well-established activation pattern, characterized by both sporadic and synchronous activity, consistent with prior observations. (A) Raster plots showing neuronal activity recorded from MS-10-derived neuronal cultures at days 21, 35, and 40. Blue dots indicate neuronal activations derived from calcium fluorescence variations (Schmitt interference spikes). The Y-axis represents the ROI index, and the X-axis denotes time in seconds. (B) Representative average fluorescence images from the neuronal activity experiments.
