# Supplementary material for: Chitinase 3‐like 1 is neurotoxic in multiple sclerosis patient‐derived cortical neurons
Source: Clin Transl Med. 2024 Dec 10;14(12):e70125. doi: 10.1002/ctm2.70125 (PMC11631566; doi:10.1002/ctm2.70125)
Supplement: Supplementary file 2 — Supporting information [file CTM2-14-e70125-s002.pdf]

Supplementary Table 1. Information on hiPSCs and RRMS patients

Unique identifier	Abbreviation in figures	Age	Gender	Ethnicity	Disease	Treatment
MS_FiPS2_R4F_10 (ESi049-A)	MS-10	44	Male	Caucasian	MS	No
MS_FiPS5_R4F_6 (ESi052-A)	MS-6	42	Female	Caucasian	MS	No

Age and treatment corresponds to the time of skin biopsies. Information adapted from Miquel-Serra et al.[24]

Supplementary Table 2. Top up-regulated (p<0.01) DEGs expressed in the CHI3L1-treated condition versus vehicle at 12 hours.

Gene Symbol	logFC	P.Value	Gene Description
**C6orf58**	0.455	0.00098947	Chromosome 6 open reading frame 58
**SCYL3***	0.560	0.00106104	SCY1 like pseudokinase 3
**FANCF**	0.459	0.00130365	Fanconi anemia complementation group F
**DEPDC4**	0.624	0.00131163	DEP domain containing 4
**AQP1***	0.425	0.00154166	Aquaporin 1
**IFNA21**	0.473	0.00172223	Interferon alpha 21
**ZNF30**	0.465	0.00279674	Zinc finger protein 30
**MLANA**	0.404	0.00330623	Melan-A
**KLRG1**	0.379	0.00348262	Killer cell lectin like receptor G1
**ABCA13***	0.466	0.00349639	ATP binding cassette subfamily A member 13
**BTBD19**	0.352	0.00407773	BTB domain containing 19
**CARMIL1**	0.347	0.00491009	Capping protein regulator and myosin 1 linker 1
**GTF3C4**	0.345	0.00508083	GTF3C subunit 4
**FAM72C**	0.338	0.00523658	Family with sequence similarity 72 member C
**RALGPS2***	0.363	0.00575197	Ral GEF with PH domain and SH3 binding motif 2
**INHBE**	0.324	0.00602996	Inhibin subunit beta E
**TMPRSS11D**	0.451	0.00608827	Transmembrane serine protease 11D
**ITPR3***	0.336	0.00613852	Inositol 1,4,5-trisphosphate receptor type 3
**SLC25A30**	0.384	0.00652596	Solute carrier family 25 member 30
**AREG**	0.336	0.00668863	Amphiregulin
**TDRD15**	0.306	0.00678009	Tudor domain containing 15
**STC1**	0.408	0.0070882	Stanniocalcin 1
**ZWILCH**	0.346	0.00731337	Zwilch, kinetochore associated
**CCL24**	0.342	0.00747813	C-C motif chemokine ligand 24
**PRAMEF20**	0.347	0.00829835	PRAME family member 20
**RAG1**	0.371	0.00850275	Recombination activating 1
**RASGEF1C**	0.333	0.00892504	RasGEF domain family member 1C
**DCDC1**	0.372	0.00904696	Doublecortin domain containing 1
**AP3B2**	0.362	0.00935164	Adaptor related protein complex 3 subunit beta 2
**EDA**	0.361	0.00971221	Ectodysplasin A

DEG: differentially expressed genes. FC: fold change. *Genes selected for RT-qPCR validation.

Supplementary Table 3. Top down-regulated (p<0.01) DEGs expressed in the CHI3L1-treated condition versus vehicle at 12 hours.

Gene Symbol	logFC	P.Value	Description
**AKR1E2***	-0.607	8.23E-05	Aldo-keto reductase family 1 member E2
**LCK***	-0.443	0.00069905	Lymphocyte-specific protein tyrosine kinase
**PDE6G**	-0.432	0.00121493	Phosphodiesterase 6G, cGMP-specific rod, gamma
**GNMT***	-0.368	0.0019048	Glycine N-methyltransferase
**BORCS6**	-0.396	0.00242834	BLOC-1-related complex subunit 6
**C6orf120**	-0.371	0.00375067	Chromosome 6 open reading frame 120
**MLKL**	-0.343	0.00478061	Mixed lineage kinase domain-like protein
**SLC38A5**	-0.348	0.00484754	Solute carrier family 38 member 5
**SMKR1**	-0.341	0.00490455	Small lysine-rich protein 1
**LOC100506124**	-0.524	0.00532272	Uncharacterized protein encoded by gene [illegible]
**TADA1**	-0.386	0.00562334	Transcriptional adaptor 1
**MRAP2**	-0.340	0.00566641	Melanocortin 2 receptor accessory protein 2
**MRM2**	-0.375	0.00566922	Mitochondrial rRNA methyltransferase 2
**NR2E1***	-0.433	0.00569621	Nuclear receptor subfamily 2 group E member 1
**LRRC66**	-0.353	0.00605167	Leucine-rich repeat-containing protein 66
**GORAB**	-0.396	0.00609479	Golgin, RAB6-interacting
**LOC101928436**	-0.425	0.00661349	Uncharacterized protein encoded by gene [illegible]
**GPATCH3**	-0.311	0.00733912	G-patch domain-containing protein 3
**TMEM88B**	-0.402	0.00740542	Transmembrane protein 88B
**AHNAK***	-0.334	0.00776027	AHNAK nucleoprotein
**PDCD7**	-0.321	0.00785004	Programmed cell death protein 7
**PABPC1L2B**	-0.457	0.00878467	Polyadenylate-binding protein 1-like 2B
**KCNK3**	-0.329	0.00884324	Potassium channel subfamily K member 3
**MEGF6**	-0.333	0.00892342	Multiple epidermal growth factor-like domains protein 6
**TADA2B**	-0.370	0.00952141	Transcriptional adapter 2-beta
**GAGE10**	-0.367	0.00955803	G antigen 10
**NEU4**	-0.342	0.00967027	Neuraminidase 4
**SOX4**	-0.517	0.00995462	SRY-box transcription factor 4
**ASB16**	-0.360	0.01008612	Ankyrin repeat and SOCS box protein 16

DEG: differentially expressed genes. FC: fold change. *Genes selected for RT-qPCR validation.

Supplementary Table 4. Top up-regulated (p<0.01) DEGs expressed in the CHI3L1-treated condition versus vehicle at 24 hours.

Gene Symbol	logFC	P.Value	Description
**TMEM161B***	0.587	0.00063593	transmembrane protein 161B
**RIOK2***	0.589	0.00065597	RIO kinase 2
**CYLC1***	0.631	0.00066315	cylicin 1
**DENND2C***	0.396	0.00073245	DENN domain containing 2C
**CD86***	0.429	0.00080773	CD86 molecule
**CFAP61***	0.729	0.00080835	cilia and flagella associated protein 61
**TPTE2***	0.519	0.00082156	transmembrane phosphoinositide 3-phosphatase and tensin homolog 2
**GRID2***	0.487	0.00102925	glutamate ionotropic receptor delta type subunit 2
**RASA2***	0.502	0.00107523	RAS p21 protein activator 2
**OR2D2***	0.418	0.00114775	olfactory receptor family 2 subfamily D member 2
**HMSD***	0.569	0.00127015	histocompatibility minor serpin domain containing
**DCDC1**	0.480	0.00137422	doublecortin domain containing 1
**CD180**	0.550	0.00138154	CD180 molecule
**CRNDE**	0.586	0.00145375	colorectal neoplasia differentially expressed
**LRRC66***	0.426	0.00146262	leucine rich repeat containing 66
**KERA**	0.578	0.00147263	Keratocan
**ERAP2***	0.490	0.00173984	endoplasmic reticulum aminopeptidase 2
**VPS54***	0.455	0.00203903	VPS54 subunit of GARP complex
**UBLCP1**	0.427	0.00228041	ubiquitin like domain containing CTD phosphatase 1
**SLC9A4**	0.406	0.00237543	solute carrier family 9 member A4
**ELF1**	0.440	0.0025381	E74 like ETS transcription factor 1
**SLC13A1**	0.461	0.00268302	solute carrier family 13 member 1
**PXMP4**	0.438	0.00276701	peroxisomal membrane protein 4
**MFAP5**	0.405	0.00283931	microfibril associated protein 5
**PROS1**	0.350	0.00285853	protein S
**UHMK1***	0.416	0.00286597	U2AF homology motif kinase 1
**OR8H3**	0.400	0.00289189	olfactory receptor family 8 subfamily H member 3
**ZNF705G**	0.660	0.002894	zinc finger protein 705G
**CDH12***	0.460	0.00309203	cadherin 12
**CALB1***	0.452	0.00324353	calbindin 1
**TSHB**	0.485	0.00338013	thyroid stimulating hormone subunit beta
**CHRNA3**	0.436	0.00355419	cholinergic receptor nicotinic alpha 3 subunit
**CCDC87**	0.374	0.00373671	coiled-coil domain containing 87
**ATG4A**	0.357	0.00388319	autophagy related 4A cysteine peptidase
**SLCO1A2**	0.541	0.00405189	solute carrier organic anion transporter family member 1A2
**GKN1**	0.366	0.00407796	gastrokine 1
**FBXO5**	0.525	0.00453722	F-box protein 5

**SKAP2**	0.355	0.00459536	src kinase associated phosphoprotein 2
**USP17L10**	0.313	0.00469516	ubiquitin specific peptidase 17 like family member 10
**OR52H1**	0.374	0.00477432	olfactory receptor family 52 subfamily H member 1
**CHRFAM7A***	0.564	0.00503393	CHRNA7 (exons 5-10) and FAM7A (exons A-E) fusion
**RHOQ-AS1**	0.391	0.00514538	RHOQ antisense RNA 1
**TRIM49B**	0.460	0.00521803	tripartite motif containing 49B
**SFR1**	0.441	0.00523994	SWI5 dependent homologous recombination repair protein 1
**ZNF404**	0.493	0.00529496	zinc finger protein 404
**CSN1S1**	0.433	0.00533453	casein alpha s1
**CPNE4**	0.386	0.00540167	copine 4
**OR4L1**	0.358	0.00553428	olfactory receptor family 4 subfamily L member 1
**OR2L13**	0.515	0.00579002	olfactory receptor family 2 subfamily L member 13
**TMEM35A**	0.346	0.00595182	transmembrane protein 35A
**ASIC5**	0.317	0.00598704	acid sensing ion channel subunit family member 5
**GNPNAT1**	0.495	0.00602116	glucosamine-phosphate N-acetyltransferase 1
**SLC25A46**	0.375	0.00624604	solute carrier family 25 member 46
**FKTN**	0.494	0.00626924	Fukutin
**SNX16**	0.469	0.0063237	sorting nexin 16
**ANO3**	0.444	0.00649407	anoctamin 3
**CGRRF1**	0.479	0.00650804	cell growth regulator with ring finger domain 1
**KCND2**	0.310	0.00653888	potassium voltage-gated channel subfamily D member 2
**TNFAIP6***	0.404	0.00661553	TNF alpha induced protein 6
**DEFB126**	0.439	0.00668255	defensin beta 126
**GPR15**	0.393	0.00684794	G protein-coupled receptor 15
**CHST15**	0.334	0.00694344	carbohydrate sulfotransferase 15
**OR5F1**	0.342	0.00696616	olfactory receptor family 5 subfamily F member 1
**CDH13**	0.447	0.00722612	cadherin 13
**EPB41L5**	0.435	0.0073211	erythrocyte membrane protein band 4.1 like 5
**CC2D2B**	0.383	0.00740025	coiled-coil and C2 domain containing 2B
**TEX10**	0.422	0.00742631	testis expressed 10
**EFCAB1**	0.396	0.00768503	Calaxin
**KLRB1**	0.346	0.00793269	killer cell lectin like receptor B1
**SV2C**	0.490	0.00797303	synaptic vesicle glycoprotein 2C
**TTC5**	0.321	0.00809866	tetratricopeptide repeat domain 5
**MTMR2***	0.537	0.00822868	myotubularin related protein 2
**TOR1AIP1**	0.339	0.00834325	torsin 1A interacting protein 1
**TEC**	0.390	0.00846082	tec protein tyrosine kinase
**SYT10***	0.336	0.00847168	synaptotagmin 10
**DUSP19**	0.390	0.00857348	dual specificity phosphatase 19
**ACOT12**	0.539	0.00878722	acyl-CoA thioesterase 12
**LINC00266-1**	0.344	0.00891099	septin 14 pseudogene 20

**OVGP1**	0.364	0.00900888	oviductal glycoprotein 1
**DCHS2**	0.345	0.00911545	dachsous cadherin-related 2
**GNPAT**	0.394	0.00913637	glyceronephosphate O-acyltransferase
**DHX35**	0.421	0.009436	DEAH-box helicase 35
**C4orf33**	0.381	0.00965493	chromosome 4 open reading frame 33
**CCDC174**	0.304	0.00967993	coiled-coil domain containing 174
**PAQR5**	0.359	0.00982047	progestin and adipoQ receptor family member 5
**SLC10A5**	0.342	0.00985671	solute carrier family 10 member 5
**NEK10**	0.457	0.00986081	NIMA related kinase 10
**ZDHHC5**	0.370	0.00994998	zinc finger DHHC-type palmitoyltransferase 5

DEG: differentially expressed genes. FC: fold change. *Genes selected for RT-qPCR validation.

Supplementary Table 5. Top down-regulated (p<0.01) DEGs expressed in the CHI3L1-treated condition versus vehicle at 24 hours.

Gene Symbol	logFC	P.Value	Description
**CGN***	-0.487	0.00027254	Cingulin
**CFAP157***	-0.547	0.00042771	cilia and flagella associated protein 157
**TSNAX-DISC1***	-0.473	0.00089222	TSNAX-DISC1 readthrough (NMD candidate)
**LRRN4***	-0.431	0.00092543	leucine rich repeat neuronal 4
**ZNF326**	-0.560	0.00125904	zinc finger protein 326
**RNF126**	-0.397	0.00150772	ring finger protein 126
**SLC5A6***	-0.400	0.00164508	solute carrier family 5 member 6
**ABT1**	-0.423	0.00177957	activator of basal transcription 1
**IKBKG**	-0.467	0.0018137	inhibitor of nuclear factor kappa B kinase regulatory subunit gamma
**PRSS35**	-0.402	0.00185114	serine protease 35
**MELTF**	-0.442	0.00186731	melanotransferrin
**CST6**	-0.413	0.00192631	cystatin E/M
**BRAT1**	-0.396	0.00216621	BRCA1 associated ATM activator 1
**TRIM66***	-0.476	0.0024558	tripartite motif containing 66
**ATP4B**	-0.411	0.00266464	ATPase H+/K+ transporting subunit beta
**ASMTL**	-0.407	0.00270428	acetylserotonin O-methyltransferase like
**A3GALT2**	-0.452	0.00281207	alpha 1.3-galactosyltransferase 2
**MLYCD**	-0.351	0.0028679	malonyl-CoA decarboxylase
**FOXP3**	-0.377	0.00311743	forkhead box P3
**KISS1R**	-0.458	0.0031541	KISS1 receptor
**COQ8B**	-0.374	0.00317763	coenzyme Q8B
**CD44***	-0.425	0.00369255	CD44 molecule (Indian blood group)
**KRT14**	-0.339	0.00385346	keratin 14
**LMNA**	-0.376	0.00392716	lamin A/C
**GNMT***	-0.335	0.00398309	glycine N-methyltransferase
**SLC15A3**	-0.389	0.0042843	solute carrier family 15 member 3
**IL9R**	-0.443	0.00440375	interleukin 9 receptor
**SIGIRR**	-0.376	0.00451397	single Ig and TIR domain containing
**FAM71E2**	-0.394	0.00483115	golgi associated RAB2 interactor family member 5B
**SEMA3G**	-0.348	0.00487679	semaphorin 3G
**FAM166C**	-0.394	0.00489115	family with sequence similarity 166 member C
**PAX2**	-0.412	0.00493368	paired box 2
**EFNA1***	-0.423	0.0050473	ephrin A1
**CRYBB1**	-0.446	0.0050667	crystallin beta B1
**C11orf86**	-0.373	0.00538842	chromosome 11 open reading frame 86
**CRYBB3**	-0.348	0.00547955	crystallin beta B3
**ZSCAN31**	-0.435	0.00560308	zinc finger and SCAN domain containing 31
**LRRC75A**	-0.367	0.00570868	leucine rich repeat containing 75A
**MPP3**	-0.317	0.00584033	MAGUK p55 scaffold protein 3
**PAFAH2**	-0.389	0.00598552	platelet activating factor acetylhydrolase 2
**LDHD**	-0.363	0.00614585	lactate dehydrogenase D
**GEMIN6**	-0.354	0.00655666	gem nuclear organelle associated protein 6
**ZNF558**	-0.320	0.00699557	zinc finger protein 558
**ZBTB12**	-0.338	0.00767668	zinc finger and BTB domain containing 12

**KLHL33**	-0.368	0.00769082	kelch like family member 33
**PLP2**	-0.443	0.007919	proteolipid protein 2
**PREX1**	-0.347	0.00791906	phosphatidylinositol-3.4.5-trisphosphate dependent Rac exchange factor 1
**TNNI1**	-0.372	0.00802321	troponin I1. slow skeletal type
**TBR1**	-0.339	0.00802408	T-box brain transcription factor 1
**AIF1L**	-0.395	0.00804562	allograft inflammatory factor 1 like
**XCR1**	-0.345	0.00809632	X-C motif chemokine receptor 1
**ZNF786**	-0.351	0.00815496	zinc finger protein 786
**GRAPL**	-0.468	0.00817596	GRB2 related adaptor protein like
**OCEL1**	-0.364	0.0082239	occludin/ELL domain containing 1
**GCAT**	-0.321	0.00862087	glycine C-acetyltransferase
**NEIL2**	-0.358	0.00873637	nei like DNA glycosylase 2
**METTL7B**	-0.461	0.00882273	thiol methyltransferase 1B
**SLC2A1**	-0.332	0.00886065	solute carrier family 2 member 1
**GPR142**	-0.330	0.0089395	G protein-coupled receptor 142
**KANK3**	-0.341	0.00901053	KN motif and ankyrin repeat domains 3
**MMP9**	-0.312	0.00902189	matrix metallopeptidase 9
**CABP5**	-0.459	0.00902857	calcium binding protein 5
**TUBA3E**	-0.301	0.00903739	tubulin alpha 3e
**NGB**	-0.344	0.00909084	Neuroglobin
**IGF2**	-0.384	0.00909385	insulin like growth factor 2
**LINC00602**	-0.419	0.00925953	long intergenic non-protein coding RNA 602
**SLC16A5**	-0.309	0.00929426	solute carrier family 16 member 5
**PTX4**	-0.368	0.00931392	pentraxin 4
**PITX3**	-0.357	0.00966985	paired like homeodomain 3
**HOXB5**	-0.341	0.00977787	homeobox B5
**CALML5**	-0.350	0.0099303	calmodulin like 5
**C16orf90**	-0.373	0.0099584	chromosome 16 open reading frame 90

DEG: differentially expressed genes. FC: fold change. *Genes selected for RT-qPCR validation.
